# Zinc finger protein 32 promotes breast cancer stem cell-like properties through directly promoting GPER transcription

**DOI:** 10.1038/s41419-018-1144-2

**Published:** 2018-11-26

**Authors:** Yanyan Li, Di Gong, Le Zhang, Hongjiang Li, Shu Zhang, Jie Zhang, Kai Li, QianWen Zheng, Gang Zhao, Yue Zhang, Yue Chen, Yafei Guo, Rong Xiang, Ping Lin, Yuquan Wei

**Affiliations:** 1 Division of Experimental Oncology, State Key Laboratory of Biotherapy and Cancer Center, West China Hospital, Sichuan University, and Collaborative Innovation Center for Biotherapy, Chengdu, China; 2 Laboratory for Experimental Oncology and Radiobiology, Center for Experimental and Molecular Medicine, Academic Medical Center and Cancer Center Amsterdam, Amsterdam, The Netherlands; 3 Department of Thyroid and Breast Surgery, West China Hospital, Sichuan University, Chengdu, China; 4 Huaxi Biobank, West China Hospital, Sichuan University, Chengdu, China; 5 Department of clinical medicine, School of Medicine, Nankai University, and Collaborative Innovation Center for Biotherapy, Tianjin, China; 6 Division of Cancer Biotherapy, State Key Laboratory of Biotherapy and Cancer Center, West China Hospital, Sichuan University, and Collaborative Innovation Center for Biotherapy, Chengdu, China

## Abstract

Breast cancer is one of the leading causes of death in women. Due to the existence of a small fraction of stem cell-like subpopulations, some breast cancer subtypes exhibit very high malignancy and resistance to multiple therapies. The underlying mechanisms of how these subtypes acquire stem cell-like properties and progress more aggressively remain largely unknown. Zinc finger protein 32 (ZNF32), a newly discovered transcription factor, has been reported to be associated with breast cancer progression. However, many questions remain about its target genes and its exact mechanisms in regulating stem cell-like properties and drug resistance. In the present study, we examined the relationship between ZNF32 and GPER, a membrane-associated estrogen receptor, and we addressed their roles in stemness regulation in human breast cancer cell lines. Our results showed that ZNF32 could induce expansion of stem cell-like subpopulations and increase drug resistance by upregulating GPER expression, in which ERK activation was also implicated. We also illustrated that ZNF32 induced GPER expression via a ZNF32 binding sequence located within the GPER promoter region. A correlation between ZNF32/GPER expression and increased tumor incidence and burden was observed in xenograft mouse models. We conclude that ZNF32 can engage GPER/ERK signalling and confer breast cancer stem cell-like properties, which may indicate poor prognosis of breast cancer patients. ZNF32 and GPER targeted therapies might provide new solutions for breast cancer treatment.

## Introduction

Metastasis development and recurrence account for most breast cancer-related deaths^1,2^. Cancer stem cells (CSCs) are responsible for tumour initiation, maintenance and metastasis^3^. A sub-population of cells characterized by their capacity to survive in non-adherent conditions and to form mammospheres has been found in breast cancer cell lines^4,5^. These groups of stem-like cells have been shown to be related to breast cancer progression. Breast cancer stem-like cells are also predicted to be responsible for tumour recurrence due to their resistance to radiotherapy, chemotherapy and endocrine therapy^6–8^.

G-protein coupled estrogen receptor (GPER or GPR30) is a novel estrogen receptor with multiple functions in diverse tissues, such as breast, uterus, ovary and brain^9,10^. It has been reported to play physiological roles in regulating the functions of the cerebral, endocrine and reproductive systems.^11,12^. GPER has also been reported to contribute to pathological responses, such as cancer cell proliferation, migration and invasion, especially during breast cancer development^11,13^. Approximately 50% of breast cancer patients have been reported to express GPER, which is consistent with the development of tamoxifen resistance^14,15^. In vivo study from transgenic mouse tumour models showed that deletion of GPER reduced the size of mammary tumours and lung metastasis, indicating that GPER is critical for breast tumour growth and distant metastasis^16^. A study of 361 breast cancer patients showed that GPER expression was associated with increased primary tumour size and the prevalence of distant metastasis^17^. Other papers have reported that GPER promotes prostate stromal cell activation and is expressed in prostate cancer stem cells^18,19^. However, the role and mechanism underlying the regulation of breast cancer stem-like cells by GPER is unclear and remains to be further elucidated.

Cys2-His2 (C2H2) zinc-finger proteins represent the largest class of putative human transcription factors and are involved in cellular processes such as proliferation, differentiation, and development;^20,21^ they are also associated with many diseases, including cancer^22^. Zinc finger protein 32 (ZNF32), a transcription factor, belongs to the Kruppel-related zinc finger family. It contains six consecutive typical C2H2 zinc-finger motifs and one degenerate C2H2 zinc-finger motif, and it may bind to DNA for transcriptional regulation. Based on our previous studies, ZNF32 protects cancer cells against oxidative stress-induced apoptosis by modulating C1QBP transcription^23^. ZNF32 could also modulate autophagy and protect breast cancer cells from stimulus-induced cell death^24^. Moreover, the mouse homologue of the ZNF32 gene, Zfp637, could markedly increase mTERT expression and telomerase activity and maintain telomere length^25^. As we recently reported, ZNF32 contributes to multidrug resistance in lung adenocarcinoma^26^. Because stem cells are predicted to be responsible for tumour resistance and to influence the effects of therapy, and since more mammospheres are observed in breast cancer cells that over-express ZNF32 during suspension culture, we hypothesized that there may be a relationship between ZNF32 and breast cancer stem cell-like properties.

Consequently, in this study, we studied the effects of ZNF32 on breast cancer stem cell populations. Then, we took advantage of a series of molecular biology and bioinformatic methods to further investigate the mechanisms of ZNF32 regulation of the stem cell-like properties of breast cancer cells. We also evaluated the impact of ZNF32/GPER in the regulation of breast cancer stem cell-like properties during tumour formation and tumour growth in mouse models. In addition, we verified that ZNF32/GPER regulates breast cancer stem cell-like properties in breast cancer tissues of patients. These previously unrecognized observations suggest that high ZNF32/GPER expression may promote breast cancer malignant progression and therapy resistance by maintaining the properties of cancer stem-like cells in breast cancer.

## Materials and methods

### Cells and regents

HEK293T, MDA-MB-231, MCF-7 and ZR-75-30 cell lines were acquired from ATCC, kept frozen immediately after receipt or used in culture less than 4 months. Maintained in complete Dulbecco’s modified Eagle’s medium (DMEM) or RPMI 1640 medium containing 10% FBS (Gibco, USA) in humidified atmosphere at 37 ^o^C, 5% CO_2,_ and regularly tested for mycoplasma to verify their negative status. Taxol (TAX) was purchased from Cytoskeleton (USA).

### Detection of ALDH1 positive cells

ALDH1 was detected using the ALDH1 PE-conjugated antibody (CST, #65583 S USA) according to the manufacturer’s protocol. Briefly, cells 1 × 10^6^/ml were fixed by methyl alcohol and were loaded with the antibody. After 60 min at 4 ^o^C, the cells were washed 3 times with cold PBS and were analyzed using flow cytometry (Beckman Coulter, USA). ALDH1 positive cells were identifiable by having greater fluorescence than control cells.

### Suspension culture and Mammosphere-forming assay

For cells suspension culture, cells were plated in 100 mm non-adhesion culture dish (JET BIOFIL) over 5 days, regularly change the medium and verified by ALDH1 expression through flow cytometry detection as above. ZNF32 and GPER expression detection in suspension or normal cultured cells were examined by flow cytometry (BD FACSAria, Franklin Lakes, NJ, USA), according to the manufacturer’s recommended protocol. ZNF32 antibody was produced and purified as previously described^27^. GPER antibody was purchased from Santa Cruz Biotechnology (Santa, sc-48525-R #B0216 USA). Rhodamine (TRITC) -conjugated AffiniPure Donkey Anti-Mouse (H + L) antibody was purchased from BBI Life Sciences (#D1100883–0100, China). FITC Conjugated Goat Anti-Rabbit Secondary Antibody was purchased from BOSTER (#BA1105, China). Individual fluorescent populations were determined using acquisition and analysis software (FlowJo 7.6).

A detailed description of the mammosphere assay protocol for the quantification of breast stem cell activity is described in a recent publication^3,28^. Briefly, cells were plated in suspension culture at 500 cells/cm^2^. Mammosphere forming was calculated by dividing the number of mammospheres (colonies > 60μm in diameter) formed by the number of cells plated and expressed as a number.

### Western blot analysis

Proteins from cells were extracted using General Protein Extraction Reagent (Bioteke, Beijing, China) supplemented with 1% protease inhibitor. 25 μg proteins were loaded and separated on 12% SDS-PAGE and then transferred electrophoretically to a 0.45μm polyvinyl difluoride membranes. The following antibodies were used: ERK antibody was purchased from Cell Signaling Technology (CST, #4695 USA). pERK antibody was purchased from Cell Signaling Technology (CST, #4370 USA). β-actin antibody was ordered from Sangon (Shanghai, #D110001 China). All above antibodies were used in a dilution ratio of 1:1000. GPER antibody was used in a dilution ratio of 1:300, was purchased from Santa Cruz Biotechnology (Santa, sc-48525-R #B0216 USA). ZNF32 antibody was produced and purified as previously described^27^, and used in a dilution of 1:50. Horseradish peroxidase-conjugated secondary antibody to rabbit IgG (1:5000, Santa), and horseradish peroxidase-conjugated secondary antibody to mouse IgG (1:8000, Santa). The membrane was developed using Immobilon™ Western Chemiluminescent HRP Substrate (Millipore). Protein expression levels were quantified and normalized to β-actin by Image J.

### Cells sensitivity to Taxol assay

Cells were plated in 96-well plates in a number of 1 × 10^4^. 24 h later, cells were treated with Taxol (TAX) (10 ng/ml or 25 ng/ml) or corresponding solvent control. The cell viability was measured by MTT (Sigma Chemicals, St. Louis, MO, USA) assay after 24 h treatment. The optical density was determined at 570 nm using the ELISA plate reader (Model 550; Bio-Rad). The results were normalized to each control. At least 3 independent experiments were ensured.

### Limiting Dilution Assay

Cells were dissociated and plated at 1, 2, 4, 8, 16 and 32 cells per well into a 96-well plate. Between 7 days after plating, the number of mammospheres found in each well was quantified under the microscope. Stem cell frequency and p-values were calculated using ELDA software^29^.

### Immunohistochemistry (IHC)

IHC was performed on breast cancer tissues. ZNF32 and GPER antibody was the same as in Western blot. ALDH1 was detected using the ALDH1 antibody (CST, #54135 USA) according to the manufacturer’s protocol. As papers described, staining intensity was scored 0 (negative), 1 (weak), 2 (moderate), and 3 (strong). Staining range was scored 0–100%^30,31^. The final score was obtained by multiplying the intensity scores with staining range. The final score of ZNF32 below 80 was considered low expression and above 175 was considered high expression.

### RNA extraction and PCR detection

Total RNA was extracted with the RNAiso plus reagent (Takara, Dalian China). cDNA synthesis was performed with All-In-One cDNA Synthesis SuperMix Kit (biotool, USA). Afterwards, real-time quantitative RT-PCR (qPCR) was performed with qPCR SYBR Green SuperMix (Takara, Dalian China). Relative mRNA expressions were normalized by β-actin. The primer sequences used are listed in Supplementary Table 1.

### Lentivirus infection and transfection

Lentivirus of shZNF32, ZNF32 over-expression, shGPER and each control were purchased from Genepharm (Shanghai, China), sequences of shRNA and negative control used are listed in Supplementary Table 2. Procedures was done followed the manufacturer’s instruction. Stable knockdown or over-expression cell lines were selected with puromycin.

Small interfering RNA (siRNA) against GPER, 5′-CUGACACCGUCGA CCAGGAdTdT-3′(siGPER 1#)/5′-GCUCUACCUAGAGCAGAAAdTdT-3′(siGPER 2#) were synthesized by Gene Pharma (Shanghai, China). pSG_5_-GPER expression plasmid was constructed by inserting expanded GPER cDNA (Gene ID: 2852) fragments into pSG5 vectors. GPER interference (designated as siGPER #1) or negative control (siNC) ZR-75-30 cells were constructed using siRNA. GPER over-expression (designated as GPER) ZR-75-30 cells were constructed using an expression plasmid (pSG5-GPER), and the control cells for the GPER over-expression group were designated as pSG5-vector. Plasmid of pEGFP-ZNF32 was stored in our lab. Transfection was mediated by Lipofectamine 2000 Transfection Reagent (Invitrogen, USA). Cells had been pre-cultured 2 h in serum-free DMEM or 1640 medium for transfection. Then plasmid was introduced into the cells using transfection reagent according to the manufacturer’s instruction.

### Chromatin immunoprecipitation (ChIP)

ChIP was performed using the EZ-Magna-ChIP TM G One-Day Chromatin Immunoprecipitation Kit (17–409 Millipore) according to the manufacturer’s instructions. Briefly, cells were cross-linked with 1% formaldehyde for 15 min at room temperature. Formaldehyde was quenched with 125 mM Glycine for 5 min, and the cells were then collected and washed. Cells and cell nuclei were lysed sequentially. The extract was sonicated and incubated overnight with anti-ZNF32 antibody and anti-IgG (ab171870, Abcam, United Kindom) at 4 °C. The bound DNA was analyzed using qPCR. The primers were listed in Supplementary Table 1.

### DNA-oligo pull down assay

A double-stranded oligonucleotide corresponding to the *GPER* promoter region (GCATTT -1623bp) with wild-type ZNF32 recognition sequences, and one with two nucleotide substitutions disrupting such sequences (as shown in Supplementary Table 3), were commercially obtained in biotinylated form. 300 μg of total protein extracts from ZR-75-30 cells with ZNF32 over-expression or not by expression plasmid were incubated with 1 μg oligonucleotides for 20 min at room temperature in 500 μl binding buffer containing 12 mM HEPES (pH 7.9), 4 mM Tris (pH 7.9), 150 mM KCl, 1 mM EDTA, 12% glycerol, 1 mM dithiothreitol and 10 μg poly (dI–dC) competitor. This was followed by addition of 30 μl streptavidin–magnetic beads (Pierce), which had been pre-treated in 500 μl binding buffer with 1 mg/ml BSA, 50 μg poly (dI–dC) and 50 μg of sheared salmon sperm DNA for 30 min at room temperature. After 2 h of incubation at 4 °C, beads were washed three times with binding buffer and the protein–DNA complexes were eluted with 30 μl × 2 SDS sample buffer at 95 ℃ for 5 min, followed by gel electrophoresis and immunoblotting analysis of the recovered material.

### Generation of GPER promoter constructs and site-directed mutagenesis

The promoter section of GCATTT region of the human GPER gene was generated by PCR using the primers in Supplementary Table 1. The PCR products were cloned with kpnI/HindIII into the luciferase-based vector pGL3-basic (Promega). Mutations in the ZNF32 binding sites were generated using the Fast Mutagenesis System (Transgen Biotech, China). The primers used were listed in Supplementary Table 1.

### Dual luciferase reporter assay

For cotransfection, HEK293T cells were transfected with 0.5 μg of GPER promoter reporter construct and 0.05 μg of pRL-TK with or without 0.5 μg of pSG5-ZNF32 using 2 μl of Lipofectamine 2000 per well in 48-well plates. 48 h post-transfection, the cells were lysed in Passive Lysis Buffer, and luciferase activity was measured in the cell lysates. The dual-luciferase reporter assay was performed according to the manufacturer’s instructions (Promega) using a Multi-Mode Microplate Reader (Synergy 2, BioTek, Winooski, VT, USA).

### Overall survival assay

The Kaplan–Meier method was used to estimate overall survival from the R2: Genomics Analysis and Visualization Platform. p-values were calculated using the log-rank test. Representative subsets for ZNF32/GPER were as follows: Mixed Tumour Breast-Clynes-121 MAS5.0-u133p2/Tumour Breast-Bergh-159 MAS5.0-u133a.

### Animals

BALB/c female nude mice were used at 6 weeks (Dashuo Chengdu). 5 × 10^6^ or indicated viable ZNF32 over-expression ZR-75-30 cells with or without GPER knockdown (named vector shGPER-NC, ZNF32 shGPER-NC, vector shGPER and ZNF32 shGPER) were subcutaneously injected to the mice respectively. 7 days after inoculation, mice were randomized into eight groups (each cell type injected mice were randomized into 2 groups, n = 10 mice/group) to receive vehicle alone (DMSO + PBS) or U0126 alone (20 mg/kg). These agents were administrated intraperitoneally every 2 days for 2 weeks. To confirm the roles of ZNF32 and GPER in tumour formation, the ZR-75-30 breast cancer cells (vector shGPER-NC, ZNF32 shGPER-NC, vector shGPER, ZNF32 shGPER) were subcutaneously injected into nude mice at different cell numbers (1 × 10^5^, 2 × 10^5^, 3 × 10^5^, 4 × 10^5^, or 5 × 10^5^). 7 days after inoculation, the mice were randomized into eight groups (the mice injected with specific cell types were randomized into 2 groups, n = 10 mice/group) to receive vehicle alone (DMSO + PBS) or U0126 alone (20 mg/kg). We considered a tumour diameter of 3 mm as tumour formation 3 weeks after subcutaneous transplantation. All experimental protocols were approved by the Committee on the Use of Live Animals in Teaching and Research of Sichuan University.

### Tissue samples from breast cancer patients

48 cases of human breast cancer samples were obtained from Huaxi Biobank of West China Hospital, patient information was shown in Supplementary table 4. The diagnosis and pathological grade of the breast cancer of all samples was confirmed by pathologist. The tissues were snap-frozen in liquid nitrogen immediately after dissection and stored in liquid nitrogen until further assessment by IHC.

The study was approved by the local Ethical Committee on Human Experimentation of West China Hospital, Chengdu, and informed written consent was acquired from all patients. Then all collected samples were eligible for experimental purpose. The methods were carried out in accordance with the approved guidelines.

### Statistical analysis

Statistical analysis was performed with SPSS 18.0 (SPSS Inc., Chicago, IL, USA). To evaluate the significant differences between two groups, the means were compared using Student’s t-test. Multiple-group comparisons were performed through one-way analysis of variance. Correlations of gene expression were determined with the Pearson coefficient. Each experiment was performed at least in triplicate, producing consistent results. *P* values < 0.05 were considered statistically significant. * indicates that the *P* values were < 0.05 and ≥ 0.01, whereas ** indicates *P* values < 0.01.

## Results

### ZNF32 induces expansion of breast cancer stem-like cell populations

**Fig. 1:**
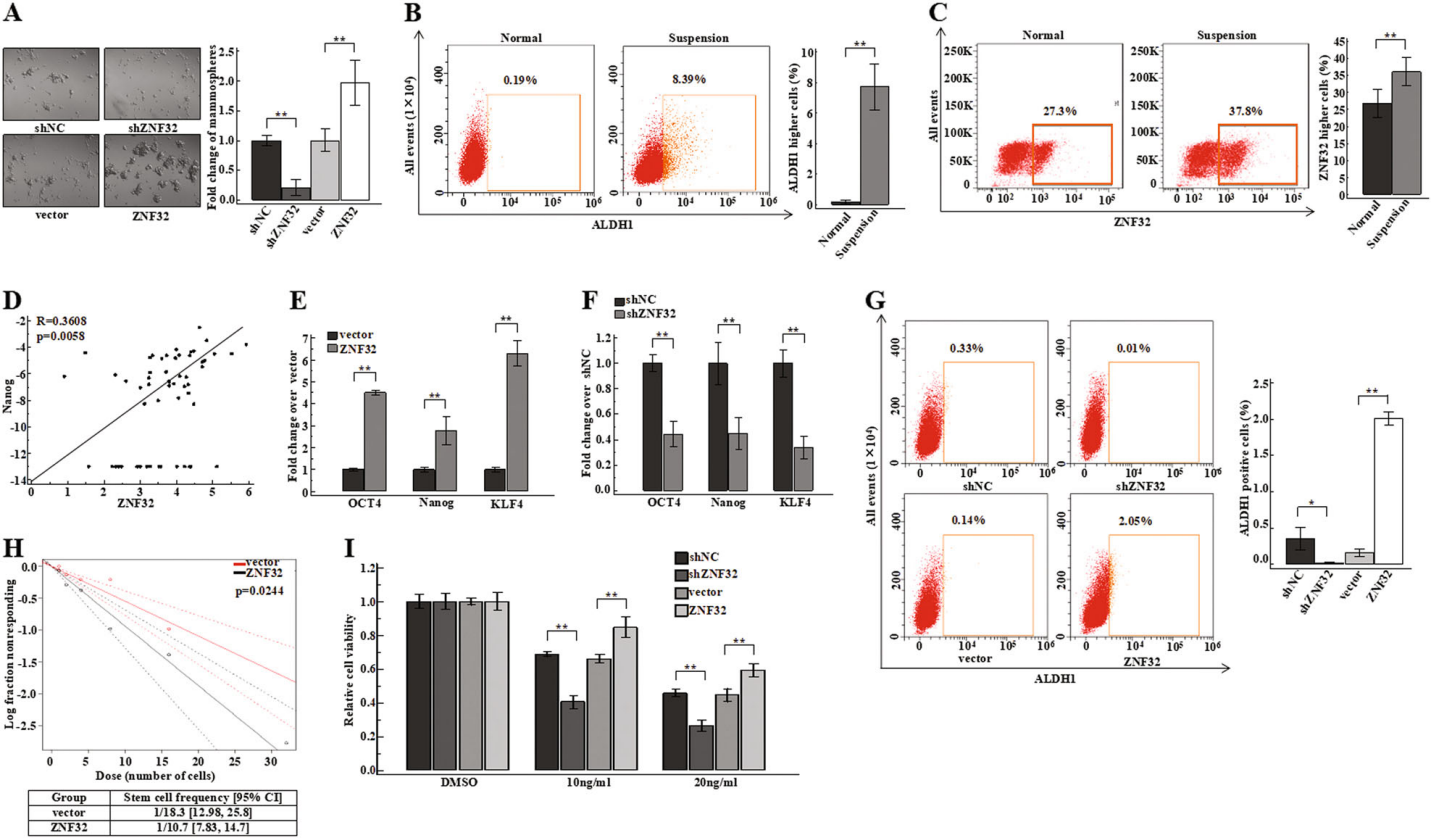
ZNF32 increases breast cancer stem-like cell populations. (**a**) Representative images of mammosphere formation, and bar graph showing the fold change in the number of mammospheres per field in the shNC, shZNF32, vector, ZNF32. shZNF32 and ZNF32 groups compared to the shNC or vector groups. (**b**) (**c**) ALDH1 and ZNF32 expression levels were measured in normal and suspension-cultured ZR-75-30 cells by flow cytometry using ALDH1^+^ or ZNF32^+^ gates based on normal cultured cells. The bar graph in the right panel shows ALDH1 or ZNF32 expression in normal or suspension-cultured cells. (**d**) Correlation analysis between *ZNF32* and *Nanog* expression levels in breast cancer cell lines using CCLE. (**e**) (**f**) *OCT4*, *Nanog* and *KLF4* expression levels were detected by qPCR in ZNF32 over-expressing or knockdown ZR-75-30 cells. (**g**) ALDH1-positive cells were detected by flow cytometry using an ALDH1^+^ gate based on shNC or vector cells. The bar graph in the right panel shows the number of ALDH1-positive cells in the shNC, shZNF32, vector, and ZNF32 ZR-75-30 groups. (**h**) Stem cell frequency was calculated using the online Extreme Limiting Dilutions Assay (ELDA) analysis program. A significant difference in stem cell frequency was detected between vector (1/18.3) and ZNF32 (1/10.7) ZR-75-30 cells. (**i**) ZR-75-30 cells with ZNF32 knockdown or over-expression were plated in 96-well plates and incubated for 24 h, and then, 10 ng/ml or 20 ng/ml Taxol or DMSO as a solvent control was added. MTT analysis was used to assess cell viability

Stable ZNF32 knockdown (referred to as shZNF32) and over-expression (referred to as ZNF32) breast cancer ZR-75-30 cells, as well as negative control cells (shNC or vector) were established as shown in Figure S1A. We found that many more mammospheres formed after ZNF32 over-expression and that fewer mammospheres were observed after ZNF32 knockdown in ZR-75-30 cells compared to vector or shNC cells (Fig. 1a). Considering that stem-like cells have a greater ability to form mammospheres, we hypothesized that ZNF32 expression may play a role in breast cancer stem cell-like properties.

Cancer cell stemness is effectively enhanced in suspension culture. We cultured breast cancer ZR-75-30 cells under normal or suspension conditions and assessed the expression of aldehyde dehydrogenase 1 (ALDH1), a candidate marker for the stem/progenitor cell population in both the normal mammary gland and mammary carcinomas^32,33^. As Fig. 1b shows, 8.39% of the ZR-75-30 cells in suspension culture had higher ALDH1 expression, while only 0.19% showed the phenotype in normal culture, indicating that the stem-like cell population successfully expands in suspension culture. Interestingly, we found that the number ZR-75-30 cells with high ZNF32 expression significantly increased in suspension culture (Fig. 1c). This observation suggested that ZNF32 might be associated with the expansion of breast cancer stem cell-like subpopulations.

We then analysed the expression of *ZNF32* and the stem cell marker *Nanog* in breast cancer cells from a database (Cancer Cell Line Encyclopedia, CCLE). Consistent with our prediction, the results showed that *ZNF32* was positively correlated with *Nanog* expression (Fig. 1d). OCT-4, Nanog and KLF4 are indispensable for the maintenance of stem cells^5,34–37^. Their expression levels were determined after ZNF32 knockdown or over-expression in ZR-75-30 cells. The expression of all these stemness-related genes was increased by ZNF32 over-expression (Fig. 1e). Meanwhile, inhibiting ZNF32 expression led to decreased expression levels of the three genes (Fig. 1f). We also evaluated the proportion of ALDH1-positive cells among ZR-75-30 cells with altered ZNF32 expression by flow cytometry. Accordingly, the percentage of ALDH1-positive cells also increased (approximately 14-fold) in ZNF32 over-expressing cells and significantly decreased (approximately 33-fold) in ZNF32 knockdown cells compared to vector or shNC cells (Fig. 1g). The percentage of ALDH1-positive cells increased in ZNF32 over-expressing cells and decreased in ZNF32 knockdown cells could also be observed in these two additional breast cancer cell lines (Fig S1B), MCF-7 (Fig S2A) and MDA-MB-231 (Fig S2B). Limiting dilution assays were used to further study the effects of ZNF32 on breast cancer stem cell frequency. The number of cells required to generate at least one tumour sphere/well was determined to be 10.7 in ZNF32 over-expressing cells and 18.3 in vector control cells (Fig. 1h). Taken together, the above results imply that ZNF32 is an important factor that could contribute to the stem cell-like properties of breast cancer cells.

Stemness of cancer cells could also equip them with more powerful capabilities to resist antitumour drug toxicity^38–40^. Taxol (TAX) is a commonly used chemotherapy drug against breast cancer^41^. We found that ZNF32 over-expression inhibited the decrease in cell viability caused by TAX compared to the vector control and that ZNF32 knockdown enhanced the effect of TAX compared to shNC (Fig. 1i). This finding suggests that ZNF32 is important for breast cancer drug resistance. Considering these results, it was reasonable to hypothesize that ZNF32 could serve as a gene for enhanced stemness in breast cancer cells.

### ZNF32 regulates GPER expression in breast cancer cells

**Fig. 2:**
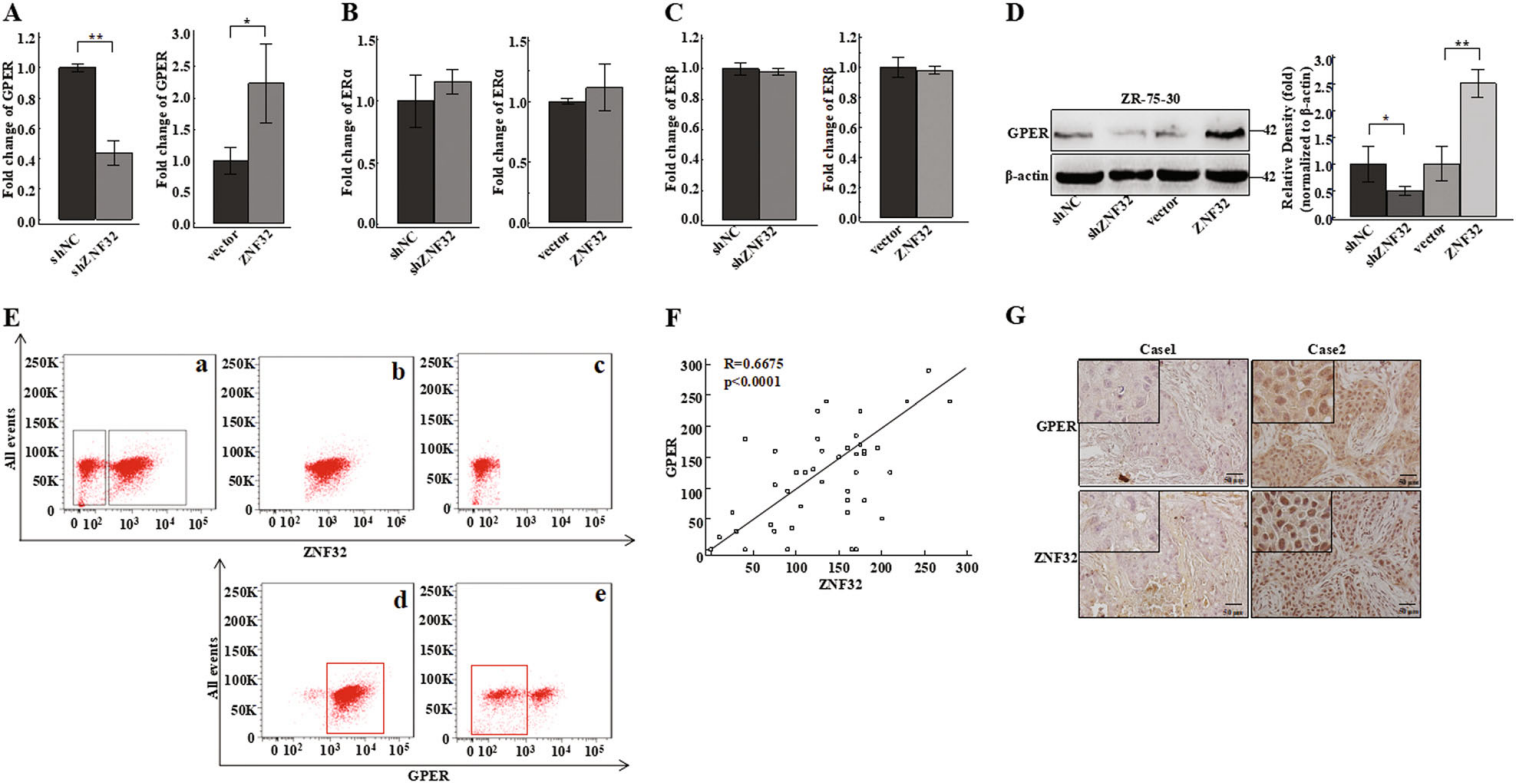
ZNF32 promotes GPER expression in breast cancer cells. (**a**) (**b**) (**c**) qPCR analysis of *GPER, ERα* and *ERβ* levels in ZNF32 knockdown or over-expressing ZR-75-30 cells. (**d**) Western blotting was used to confirm the effect of ZNF32 knockdown or over-expression on GPER expression in ZR-75-30 cells, and the right panels show the quantification of 3 independent experiments. (**e**) Suspension-cultured ZR-75-30 cells were incubated with mouse anti-ZNF32 and rabbit anti-GPER antibodies and then with anti-mouse or anti-rabbit rhodamine or FITC-conjugated secondary antibodies. As detected by flow cytometry using a ZNF32^+^ gate based on ZR-75-30 cells (**a**), the ZNF32 higher expressing (**b**) and lower expressing (**c**) cells are shown using GPER^+^ gating (**d**) (**e**). (**f**) Correlation analysis between ZNF32 and GPER expression levels in breast cancer patient samples. (**g**) Representative ZNF32 and GPER expression levels in breast cancer samples were detected by IHC

Because of the close relationship between estrogen receptors and breast cancer cell stemness and because estrogen receptors participate in breast cancer development and drug resistance^14,15,17,18^. The ZNF32 binding sequence was found in the promoter regions of several estrogen receptor genes (*ERα*, *ERβ* and *GPER*). We next measured the expression levels of these three genes in ZNF32 stable knockdown or over-expressing ZR-75-30 cells to explore whether ZNF32 can regulate their expression. Surprisingly, after ZNF32 down-regulation or up-regulation, only the mRNA the level of *GPER* (Fig. 2a) changed, while the expression of *ERα* (Fig. 2b) and *ERβ* (Fig. 2c) was unchanged. We also verified these effects in MCF-7 and MDA-MB-231 (Fig S3A). GPER protein level showed significant down-regulation in ZNF32 stable knockdown cells and up-regulation in ZNF32 over-expressing cells compared to shNC or vector control cells (Fig. 2d S3B).

To further validate the regulation of GPER by ZNF32, we measured ZNF32 and GPER expression in suspension-cultured ZR-75-30 cells using flow cytometry. The cells (Fig. 2e a) were divided into high ZNF32-expressing cells (Fig. 2e b) and lower ZNF32-expressing cells (Fig. 2e c), and then, we divided the two groups of cells based on the GPER expression level. The results showed that cells with high ZNF32 expression showed higher GPER expression (Fig. 2e d) and that cells with lower ZNF32 expression showed low GPER expression (Fig. 2e e). These results suggested that ZNF32 could indeed promote GPER expression in breast cancer cells.

Next, we measured ZNF32 and GPER expression in breast cancer tissues using IHC. Coincidently, the results showed a positive correlation between ZNF32 expression and GPER expression (Fig. 2f), and representative figures are shown in Fig. 2g.

### ZNF32 directly binds to the GPER promoter to transcriptionally regulates *GPER* in breast cancer cells

**Fig. 3:**
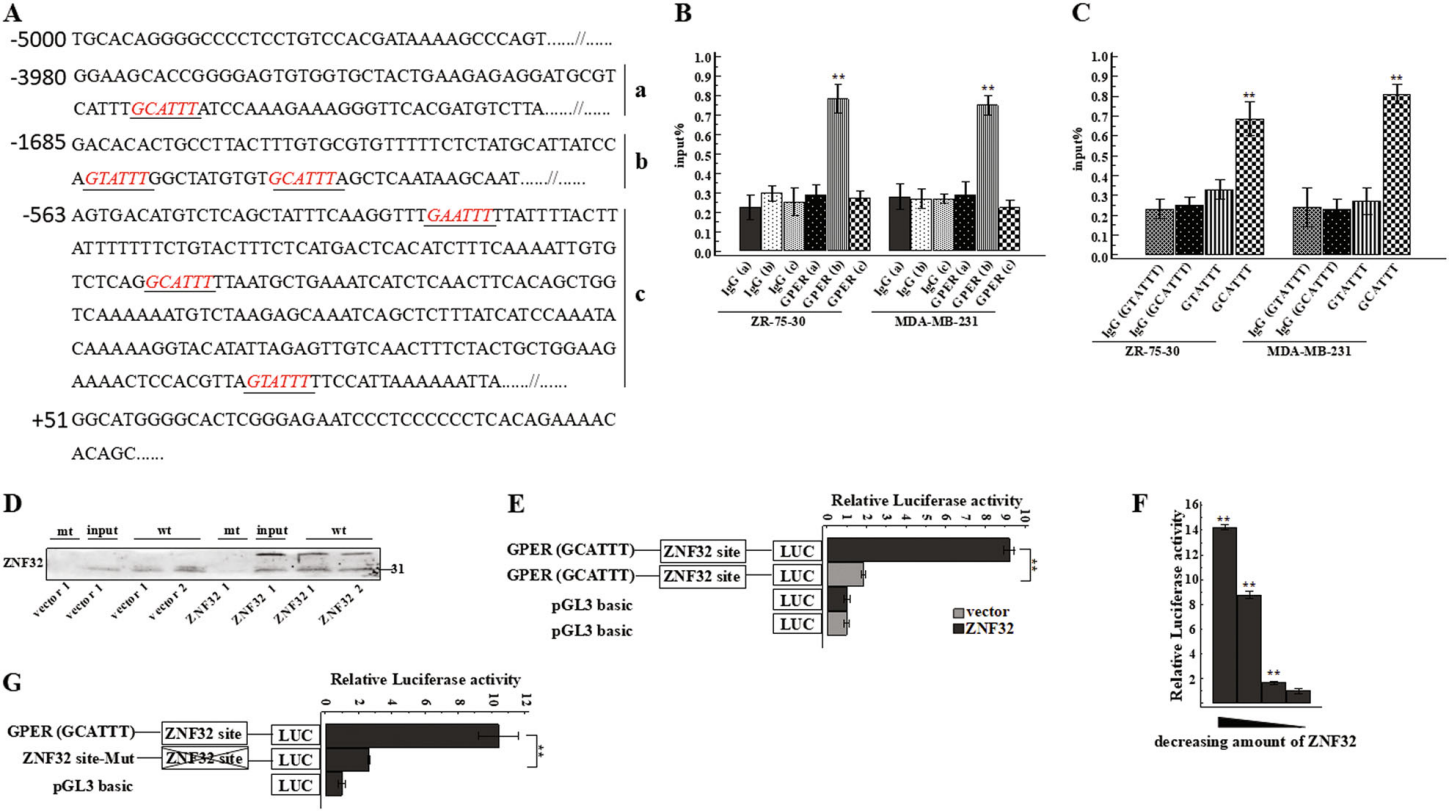
ZNF32 directly regulates GPER expression by binding to the *GPER* promoter. (**a**) Schematic representation of the six ZNF32-binding sites in the *GPER* promoter (−5000 bp to + 100 bp). The consensus sequences for the predicted ZNF32 sites are shown in red italics. (**b**) (**c**) DNA fragments from ZR-75-30 and MDA-MB-231 cells immunoprecipitated with ZNF32-specific antibodies and analysed via qPCR using the indicated primers. (**d**) ZR-75-30 cell extracts with or without ZNF32 over-expression (ZNF32) were incubated with biotinylated oligonucleotides with tandem ZNF32 binding sites from the *GPER* gene (wt) or oligonucleotides with site disruption (mt). Pull down samples were immunoblotted with antibodies against ZNF32. (**e**) (**f**) (**g**) HEK293T cells were transiently transfected with the indicated constructs and then analysed using a dual luciferase reporter assay

As ZNF32 is a transcription factor in the zinc finger superfamily, we wondered whether ZNF32 can directly interact with the *GPER* promoter and regulate GPER expression at the transcriptional level. ZNF32 specific DNA binding sequence had been revealed as we previously described^42^. First, the *GPER* promoter (-5000 bp to + 100 bp) was analysed, and six potential ZNF32-binding sequences, G(A/T/C)ATTT, were found (Fig. 3a). Next, ChIP analysis was performed in ZR-75-30 and MDA-MB-231 cells. We designed three pairs of primers (S table 1) to detect the three binding fragments containing the ZNF32-specific binding sequences (Fig. 3a a b c area). One fragment, which contained two ZNF32-binding sequences (−1639 bp/−1623 bp, GTATTT/GCATTT), was significantly enriched after co-precipitation with an antibody against ZNF32 in both cell lines (Fig. 3b). However, for the other two fragments, no binding signal could be detected (Fig. 3b). To narrow down and eventually determine the exact functional binding sequence of ZNF32 in this one reactive fragment, we designed two additional primer pairs (S table 1). The products of each pair of primers contained one ZNF32-specific binding sequence. ChIP analysis showed that ZNF32 could only bind to the GCATTT sequence in the *GPER* promoter at the −1623 bp position (Fig. 3c). This result implied that ZNF32 directly interacts with the GPER promoter at the −1623 bp GCATTT and enhances the expression of the *GPER* gene. To further confirm that this binding sequence in the *GPER* promoter is ZNF32 specific, a DNA-oligo pull-down assay was carried out in ZR-75-30 cells with a specifically synthesized oligonucleotide containing the ZNF32-binding sequence (−1623 bp GCATTT) of the *GPER* gene and a synthesized control oligonucleotide containing two nucleotide changes that could disrupt ZNF32 recognition. Accordingly, ZNF32 could be effectively pulled down by only the specific oligonucleotide and not by the control oligonucleotide containing the nucleotide changes (Fig. 3d). In addition, a dual-luciferase reporter assay was performed, and we found that the transcriptional activity of the GPER promoter (GCATTT) was significantly increased after transfection with a ZNF32-expressing plasmid (Fig. 3e). Increased levels of ZNF32 led to a dose-dependent increase in GPER transcription in HEK293T cells (Fig. 3f). To further confirm the direct binding of ZNF32 to the GPER promoter, we engineered a mutation at the putative ZNF32-binding site in the GPER promoter (GCATTT). As shown in Fig. 3g, compared with wild-type GPER (GCATTT), mutant GPER showed decreased GPER promoter-associated luciferase activity due to functional disruption of the identified ZNF32 response elements. Taken together, these results show that ZNF32 could directly bind to the *GPER* promoter at a ZNF32-specific binding site (−1623 bp GCATTT) to regulate GPER transcription.

### GPER expression promotes breast cancer stem-like cells expansion

**Fig. 4:**
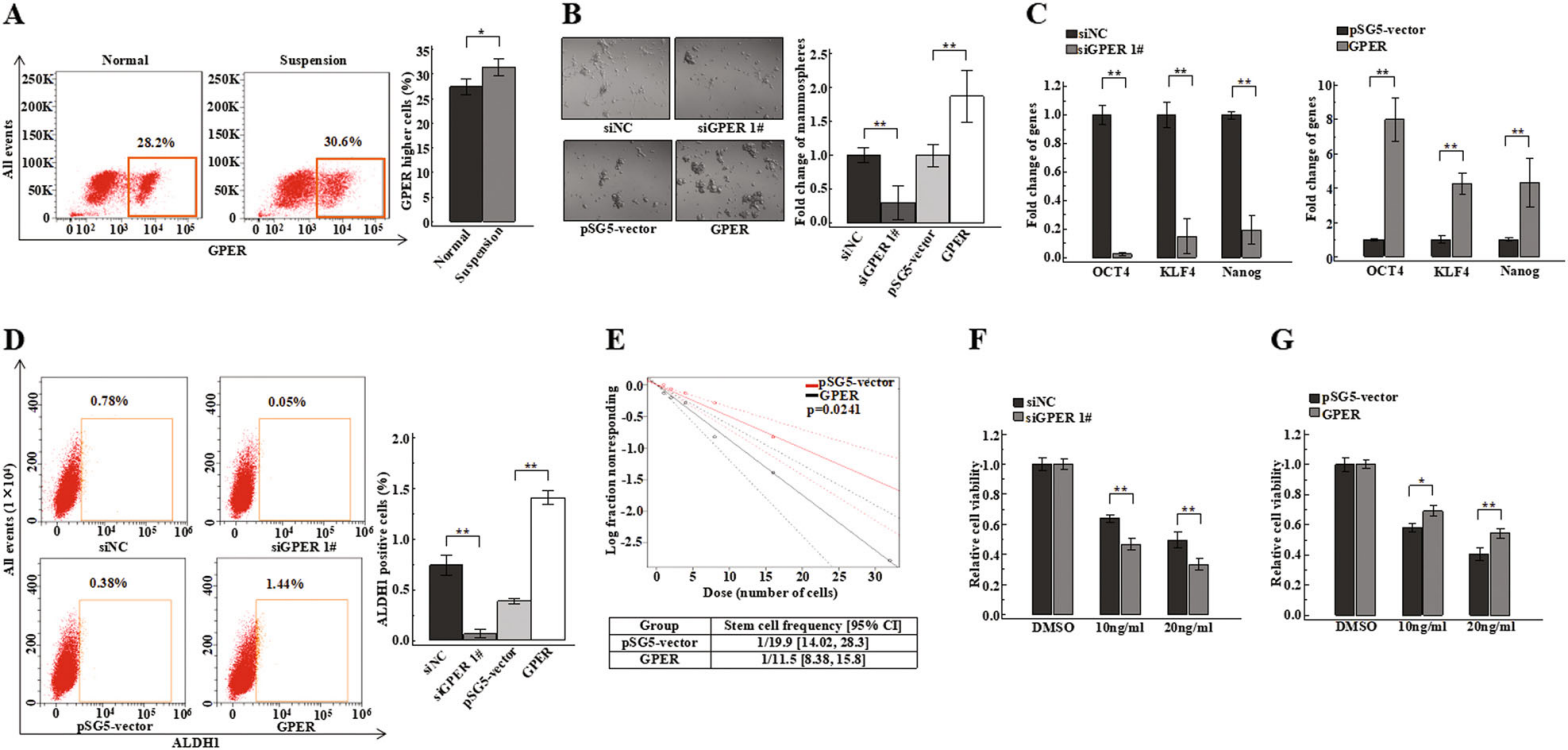
GPER could increase the stem cell-like properties of breast cancer cells. (**a**) GPER expression was measured in normal and suspension-cultured ZR-75-30 cells by flow cytometry using GPER^+^ gates based on normal cultured cells. The bar graph in the right panel shows GPER expression in normal or suspension-cultured cells. (**b**) Representative images of mammosphere formation, and bar graph showing the fold change in the number of mammospheres per field in the siNC, siGPER 1#, pSG5-vector, GPER. siGPER 1# and GPER groups compared to the siNC or pSG5-vector groups. (**c**) *OCT4*, *KLF4* and *Nanog* expression levels were detected by qPCR in GPER over-expressing or knockdown ZR-75-30 cells. (**d**) ALDH1-positive cells were detected by flow cytometry using an ALDH1^+^ gate based on siNC or pSG5-vector cells. The bar graph in the right panel shows the number of ALDH1-positive cells in the siNC, siGPER 1#, pSG5-vector, GPER ZR-75-30 groups. (**e**) Stem cell frequency was calculated using the online Extreme Limiting Dilutions Assay (ELDA) analysis program. A significant difference in stem cell frequency was detected between pSG5-vector (1/19.9) and GPER (1/11.5) ZR-75-30 cells. (**f**) (**g**) ZR-75-30 cells with GPER knockdown or over-expression were plated in 96-well plates and incubated for 24 h, and then, 10 ng/ml or 20 ng/ml Taxol or DMSO as a solvent control was added. MTT analysis was used to assess cell viability

Since a number of studies have shown that GPER might be a regulator of prostate cancer cell stemness and to be relevant for breast cancer tumour size and distant metastasis^17,19^, we further explored the relationship between GPER expression and stem cell-like properties in breast cancer. First, we measured GPER expression in normal or suspension-cultured ZR-75-30 cells by flow cytometry. We observed that GPER-high cells were also significantly increased in suspension-cultured ZR-75-30 cells (Fig. 4a). We found that GPER is an important factor that could contribute to the stem cell-like properties of breast cancer cells too (Fig S1C Fig. 4b–g Fig S4A). To avoid off-target effects of GPER siRNA, we synthesized another siRNA against GPER (siGPER #2). We found that this siRNA showed a similar effect as siGPER #1 on breast cancer stem cell-like properties (Fig S1D, S4A-E). In summary, our results indicated that GPER confers breast cancer cells with more stem cell-like properties.

### GPER is essential for ZNF32-induced breast cancer stem cell-like properties

**Fig. 5:**
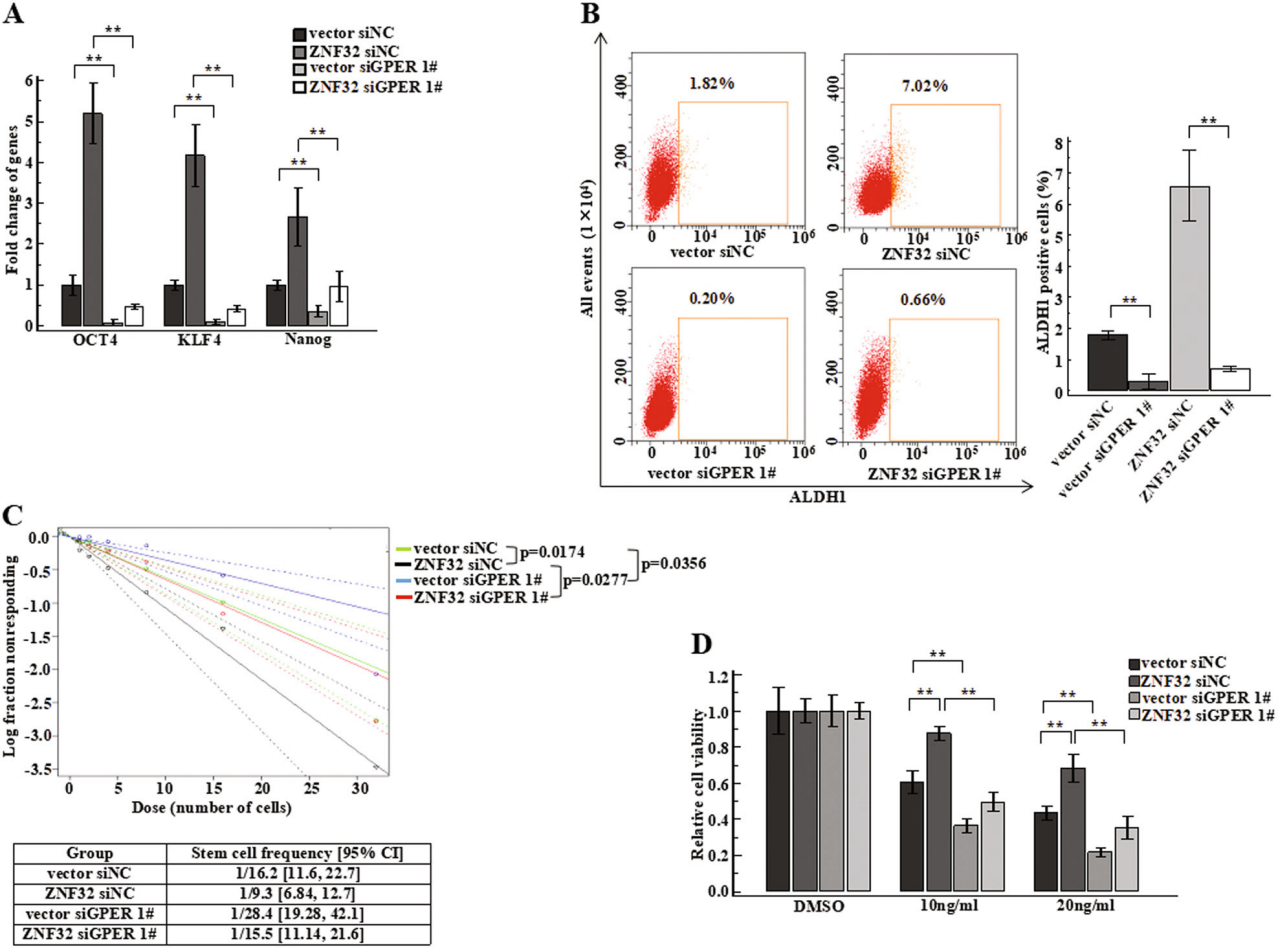
ZNF32 promotes breast cancer stem cell-like properties associated with drug resistance partially by up-regulating GPER expression. (**a**) *OCT4*, *KLF4* and *Nanog* gene expression were measured by qPCR in ZNF32 over-expressing ZR-75-30 cells with or without GPER knockdown. (**b**) ALDH1 positive cells were detected by flow cytometry, showed using ALDH1^+^ gate based on ZR-75-30 cells vector siNC. Bar graph in the right panel was ALDH1 positive cells in vector siNC, ZNF32 siNC, vector siGPER 1#, ZNF32 siGPER 1# cells. (**c**) The stem cell frequencies were calculated using the online Extreme Limiting Dilutions Assay (ELDA) analysis program. Significant differences in stem cell frequencies were detected between vector siNC (1/16.2), ZNF32 siNC (1/9.3), vector siGPER 1# (1/28.4), and ZNF32 siGPER 1# (1/15.5) cells. (**d**) ZR-75-30 cells with ZNF32 over-expression were plated in 96-well plates, and GPER expression was knocked down using siRNA. After 24 h of incubation, 10 ng/ml or 20 ng/ml Taxol or DMSO (control) was added, and MTT analysis was used to detect cell viability

Because of the similar functions of ZNF32 and GPER in breast cancer cell stemness regulation and since ZNF32 regulated GPER expression at the transcriptional level, we hypothesized that ZNF32 might affect the stem cell-like properties of the breast cancer cells through a GPER-dependent pathway. To further address this question, we first knocked down GPER expression by specific siRNA in ZNF32-over-expressing ZR-75-30 cells. ZNF32 over-expression-induced GPER expression was significantly down-regulated compared with control (Fig S1E). qRT-PCR measurement revealed that the up-regulation of *OCT4, KLF4* and *Nanog* expression due to ZNF32 over-expression could be decreased by knockdown of GPER expression (Fig. 5a). In addition, the percentage of ALDH1-positive cells in ZNF32 over-expression ZR-75-30 cells was also dramatically decreased when GPER expression was inhibited (Fig. 5b). GPER knockdown could significantly decrease the number of colonies that were induced by ZNF32 (Fig. 5c). Taken together, GPER expression was shown to be critical for the stem cell-like properties of breast cancer cells induced by ZNF32 over-expression.

For further analyse the significance of GPER in breast cancer stem-like cell populations caused by ZNF32 overexpression, we evaluated their role in Taxol-associated cell viability. Compared with vector cells, ZNF32 over-expression cells showed resistance to TAX treatment, and this effect could be counteracted by GPER interference (Fig. 5d). These results suggested that breast cancer cells with increased ZNF32 expression could acquire stronger drug resistance via the GPER axis. siGPER #2 conferred the same phenotype as siGPER #1 (Fig S1F, S4F G). This finding suggests that GPER is essential for ZNF32-induced breast cancer stem cell-like properties.

### Breast cancer stem cell-like properties are regulated by ZNF32 through GPER and the associated ERK transduction pathway

**Fig. 6:**
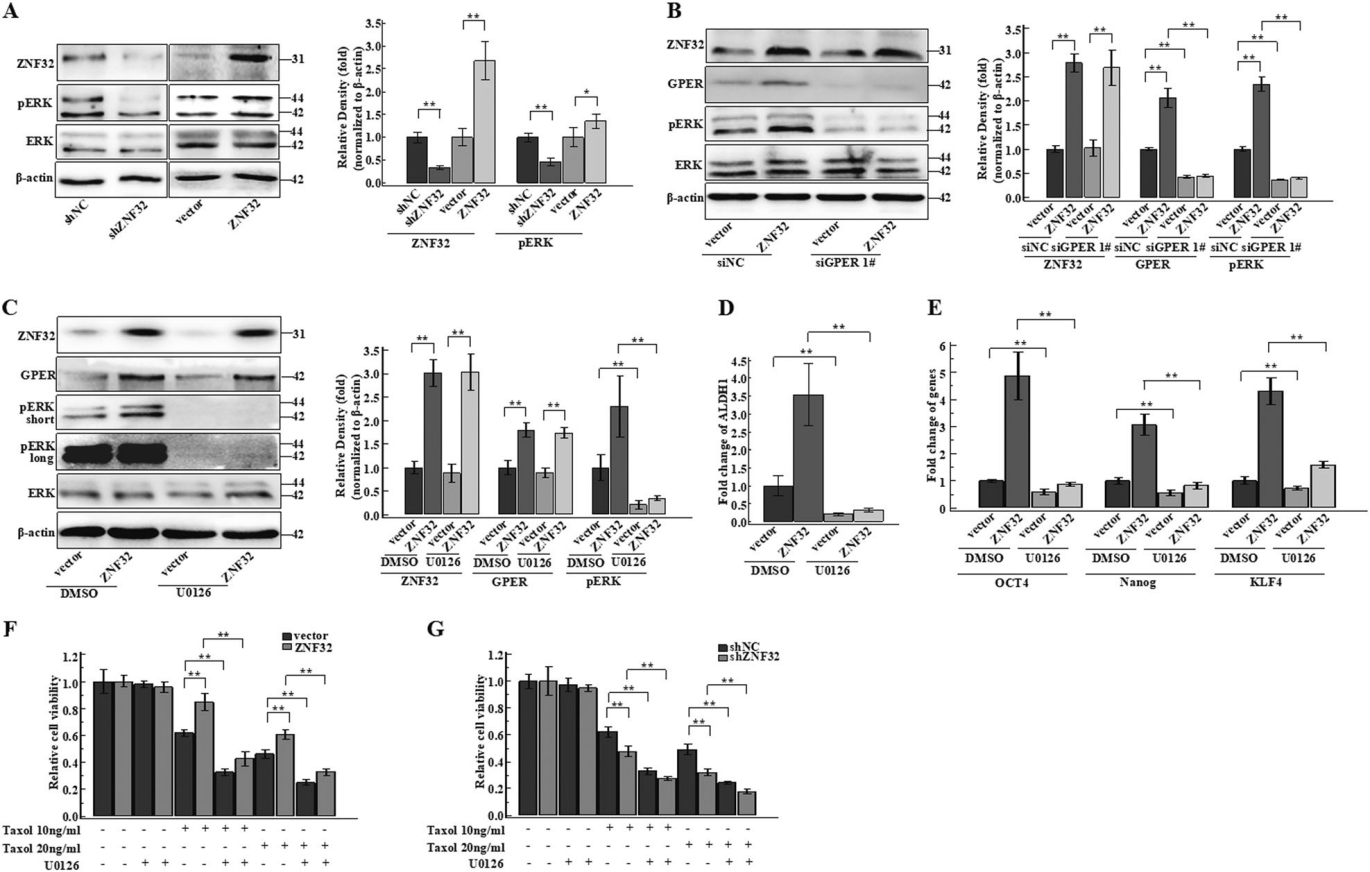
ZNF32 and GPER regulate breast cancer stem cell populations by activating the ERK pathway. (**a**) Western analysis was used to detect ERK activation. Quantification of ZNF32 and pERK expression in 3 independent experiments. (**b**) Western analysis was used to detect ERK activation in ZNF32 over-expressing cells with or without GPER interference. Quantification of ZNF32 and pERK expression levels from 3 independent experiments. (**c**) ERK inhibitor U0126 (20 μM) or DMSO (control) was added to ZR-75-30 cells over-expressing ZNF32, followed by incubation for 24 h. Western analysis was used to detect ERK activation. Quantification of ZNF32 and pERK levels from 3 independent experiments. (**d**) qPCR analysis was used to measured *ALDH1* gene expression in ZNF32 over-expressing or vector control ZR-75-30 cells treated with or without U0126 or DMSO for 24 h. (**e**) qPCR analysis was used to detect *OCT4*, *Nanog* and *KLF4* gene expression in ZNF32 over-expressing or vector control ZR-75-30 cells treated with or without U0126 or DMSO for 24 h. (**f**) (**g**) ZR-75-30 cells with ZNF32 over-expression or knockdown were plated in 96-well plates. After 24 h of incubation, pretreatment with 20 μM U0126 was performed for 2 h before the addition of 10 ng/ml or 20 ng/ml Taxol or DMSO (control), and MTT analysis was used to detect cell viability

It has been reported that ligands binding to GPER could induce the release of membrane-tethered heparin-bound epidermal growth factor, which binds to and activates the epidermal growth factor receptor (EGFR)^43,44^. As reported, the transactivation of EGFR could stimulate a transduction network that might further induce ERK activation^45,46^. ERK was demonstrated to activate transcription of the *ALDH1* gene and to induce stemness^47^. We next measured ERK phosphorylation in our ZR-75-30 cell model. The results showed that ZNF32 over-expression could promote ERK activation and that ZNF32 knockdown resulted in the opposite result (compared to thevector or shNC) (Fig. 6a). To make sure that ZNF32 regulation of ERK signalling is via GPER, we measured ERK phosphorylation in ZNF32 over-expressing cells with or without GPER interference. The results showed that the increased pERK level due to ZNF32 over-expression was significantly down-regulated by GPER interference (Fig. 6b). Furthermore, GPER knockdown decreased the difference in pERK levels between ZNF32 over-expressing and negative control cells. To further demonstrate the role of the ERK signalling pathway in ZNF32/GPER-regulated stem cell-like properties, we next used the ERK phosphorylation inhibitor U0126. We found that ERK phosphorylation upon ZNF32 over-expression was abrogated in the presence of U0126. In contrast, ZNF32 and GPER expression levels were not affected (Fig. 6c). As ERK transduction signalling triggers *ALDH1* gene expression, we determined the occurrence of this response with the ERK inhibitor U0126 in ZNF32 over-expressing and vector ZR-75-30 cells. In agreement with these data, ZNF32 over-expression induced a rapid increase in *ALDH1* gene expression compared to control, which was rescued by inhibition of ERK phosphorylation (Fig. 6d). Further supporting these results, the up-regulation of the stem cell markers *OCT4*, *Nanog* and *KLF4* induced by ZNF32 over-expression was abolished by ERK inhibition (Fig. 6e). To further study the role of ERK signalling in ZNF32/GPER-induced breast cancer stem cell-like properties, Taxol-associated breast cancer cell viability was evaluated in the presence of U0126. We found that ZNF32 over-expression inhibited the decreased cell viability caused by TAX compared to vector control and that ZNF32 knockdown enhanced the effect of TAX compared to shNC (Fig. 6f, g). The ERK inhibitor U0126 caused the cells to be more sensitive to Taxol and diminished the difference in both ZNF32 knockdown and over-expressing cells compared with the respective control cells (Fig 6f, g). Taken together, these results suggest that GPER, along with the ERK signal transduction pathway, mediates the breast cancer stem cell-like properties induced by ZNF32.

### ZNF32 and GPER promote tumour formation and tumour growth in vivo

**Fig. 7:**
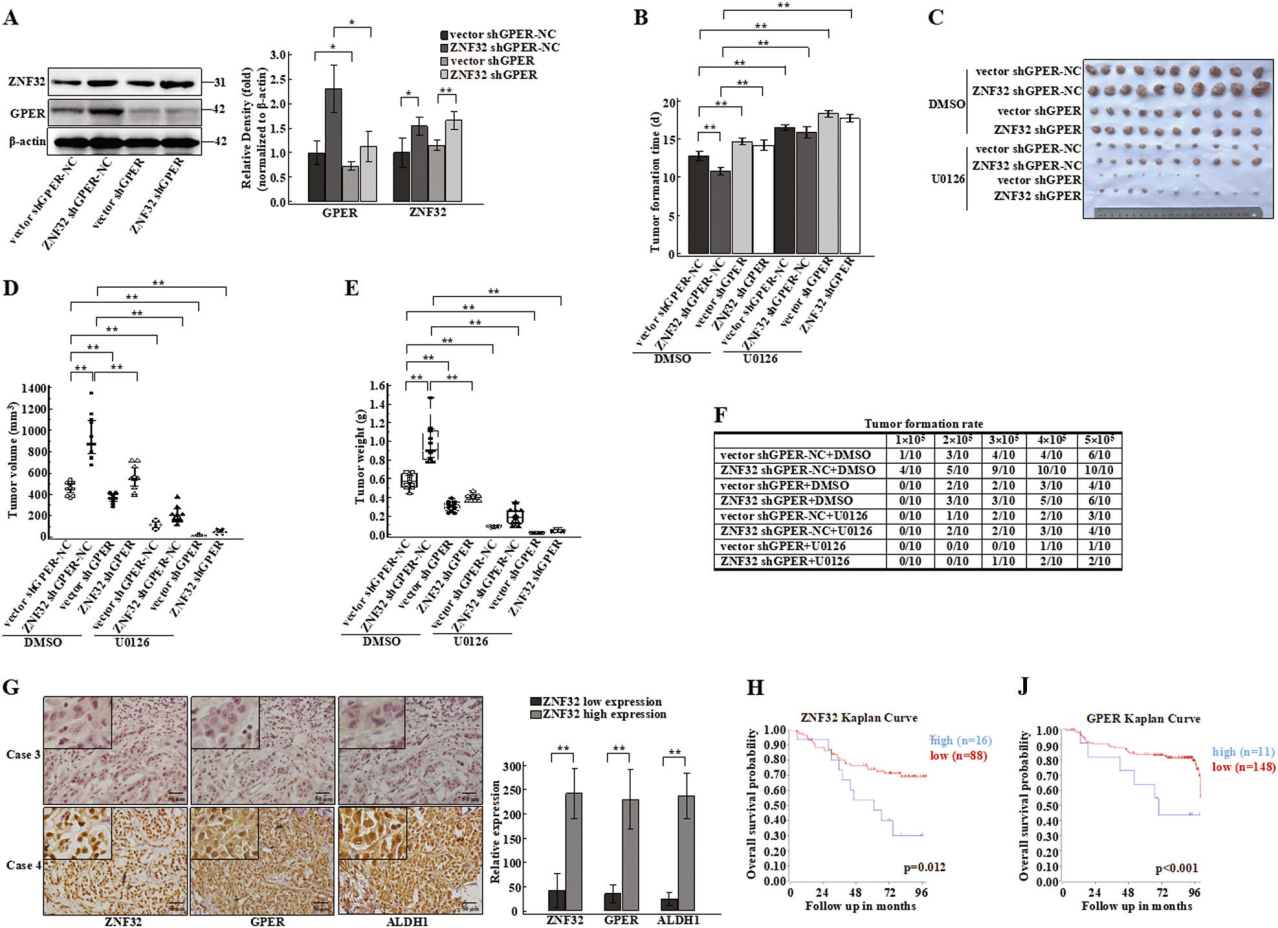
The roles of ZNF32 and GPER in tumour formation in xenografts. (**a**) Western analysis was used to detect the GPER knockdown efficiency in ZNF32 over-expressing ZR-75-30 cells. Quantification of 3 independent experiments is shown in the right panel. A total of 5 × 10^6^ viable cells were implanted subcutaneously into nude mice. 7 days after inoculation, the mice received U0126 or vehicle. A tumour diameter of 3 mm was considered tumour formation, and the tumour formation times were recorded (**b**). Tumours were collected 3 weeks after implantation (**c**). Tumour volume (**d**) and tumour weight (**e**) were calculated. (**f**) A total of 1 × 10^5^, 2 × 10^5^, 3 × 10^5^, 4 × 10^5^ or 5 × 10^5^ viable cells were implanted subcutaneously into nude mice. 7 days after inoculation, the mice received vehicle or U0126. A tumour diameter of 3 mm was considered tumour formation, and the tumour formation was recorded 3 weeks after subcutaneous transplantation. (**g**) Representative images of ZNF32, GPER and ALDH1 expression in breast cancer samples as detected by IHC. Quantification of detected result is shown in the right panel. (H) (I) Overall survival probability of breast cancer patients with the indicated gene expression levels

To examine the effects of ZNF32/GPER-regulated breast cancer cell stemness on tumour formation and tumour growth in vivo, we established stable knockdown of GPER expression in ZNF32 over-expressing ZR-75-30 cells (Fig. 7a). 7 days after inoculation, the mice were randomized into eight groups to receive U0126 or not. We observed the condition of the mice every day and considered a tumour diameter of 3 mm as tumour formation. The ZNF32 over-expression group (ZNF32 shGPER-NC + DMSO) showed the earliest tumour formation (Fig. 7b). Interference against GPER expression alone (vector shGPER + DMSO) or knockdown of its expression in ZNF32 over-expressing cells (ZNF32 shGPER + DMSO) delayed tumour formation (compared to vector shGPER-NC + DMSO or ZNF32 shGPER-NC + DMSO groups) (Fig. 7b). Interestingly, mice that received the ERK inhibitor U0126 had further delays in tumour formation (Fig. 7b). Tumour tissues were collected at the end of third week following post-subcutaneous transplantation (Fig. 7c). There was a significant increase in tumour volume in the ZNF32-over-expression group (ZNF32 shGPER-NC + DMSO) compared to the control breast cancer cell groups (vector shGPER-NC + DMSO) (Fig. 7c, d) and a significant decrease in tumour volume in the GPER-knockdown group (ZNF32 shGPER + DMSO/vector shGPER + DMSO) compared to the control groups (ZNF32 shGPER-NC + DMSO/vector shGPER-NC + DMSO) (Fig. 7c, d). Furthermore, mice that received U0126 showed a further decrease in tumour volume (Fig. 7c, d). The tumour weight trend was the same as the volume trend (Fig. 7e).

We also evaluated the roles of ZNF32 and GPER in tumour formation. The ZNF32-over-expression group (ZNF32 shGPER-NC + DMSO) only required 1 × 10^5^ cells to form tumours. With the same cell numbers, the ZNF32-over-expression group showed the highest tumour incidence. Knockdown of GPER expression (ZNF32 shGPER + DMSO/vector shGPER + DMSO) led to a requirement for high cell numbers for tumour formation, and the tumour incidence was lower with the same cell numbers compared to the control groups (ZNF32 shGPER-NC + DMSO/vector shGPER-NC + DMSO) (Fig. 7f). When mice received the ERK inhibitor U0126, the tumour formation rate was further decreased (Fig. 7f). In conclusion, the ZNF32/GPER/ERK axis was positively associated with breast cancer development in vivo.

To examine the clinical relevance of ZNF32 and GPER expression in breast cancers, we measured the expression of GPER and ALDH1 in ZNF32-high or ZNF32-low expression breast cancer samples using IHC. Consistent with the previous results, the ALDH1 and GPER expression level were higher in samples with high expression of ZNF32 and lower in samples with low ZNF32 expression (Fig. 7g). We then analysed publicly available patient datasets, found that elevated ZNF32 and GPER expression was significantly associated with a higher risk of death in patients with breast cancer (Fig. 7h, i). This finding suggests that ZNF32 and GPER expression levels are potential predictors of breast cancer progression and development.

## Discussion

The intracellular mechanisms regulating breast cancer stem cell-like populations have been gaining attention. The roles of ZNF32 and GPER in mediating proliferation and drug resistance have been recently highlighted^26,48,49^. However, the role of ZNF32 and GPER in breast cancer stem cell-like properties and their functional significance have remained rather enigmatic. In this study, ZNF32 showed the ability to facilitate mammosphere formation and the expression of ALDH1, *OCT4*, *Nanog* and *KLF4* in breast cancer cells. In addition, our results prove that ZNF32 and ALDH1 have the same expression trend in patient breast cancer tissues. The enhanced expression of ZNF32 contributes to tumour formation and tumour growth in breast cancer xenografts. Consistently, high ZNF32 expression predicted poor survival (Fig. 7g). These results demonstrated that ZNF32 is involved in promoting stem cell-like properties of breast cancer and breast cancer progression.

**Fig. 8:**
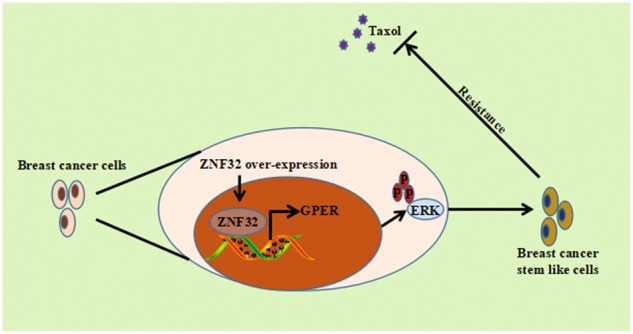
ZNF32 and GPER on the breast cancer stemness landscape toward breast tumor progression and drug resistance

GPER expression was shown to be positively correlated with breast cancer stem cell-like properties and that ZNF32 regulates GPER expression. GPER mediates the stem cell-like properties of breast cancer cells induced by ZNF32, as demonstrated by our silencing experiment. ERK signalling, which is the downstream effector of GPER, is activated and subsequently stimulates expression of the *ALDH1* gene to induce breast cancer stem cell-like properties (Fig. 8). In agreement with these findings, the pERK inhibitor U0126 reduced the increases in both the stemness index and *ALDH1*, *OCT4*, *Nanog* and *KLF4* gene expression in breast cancer cells.

High expression of GPER protein was reported to correlate with poor prognosis in breast carcinoma^17^. A study investigating 321 invasive and 40 intraductal breast tumours showed associations between GPER expression and tumour size and the presence of distant metastasis; however, it did not evaluate possible associations with overall survival^17^. Furthermore, GPER expression was assessed in 323 breast cancer patients with a validation cohort of 103 patients, which showed associations between GPER expression and lymph node status and demonstrated an association between high GPER expression and adverse relapse-free survival; however, no association was observed for overall survival^50^. Interestingly, we were also able to demonstrate that high GPER expression was significantly associated with adverse overall survival in breast cancer (Fig. 7h). These findings suggest that high levels of ZNF32 in breast cancer may result in strong GPER activation, which in turn promotes tumour progression. Previously published studies have investigated GPER expression in patient samples to show a number of associations with clinicopathological variables, however the results from these have not always been in agreement. One of the largest studies to date investigated 981 primary invasive breast carcinomas, demonstrated that low expression of GPER was significantly associated with adverse patient survival^51^. Other studies have also investigated GPER expression, demonstrated that low GPER protein and mRNA expression is associated with adverse survival in a large cohort of breast cancer patients^52^. Associations between GPER expression and status of ER, PgR, HER2 or Lymph node status have been described in some studies^52^. In addition, the types of breast cancer and status whether breast cancer patients are postmenopausal may affect the prognosis of GPER expression to breast cancer patients.

As a transcription factor, ZNF32 plays a critical role in gene regulation. Our previous study indicated that ZNF32 regulates the transcription of C1QBP and mTERT. Here, we demonstrated that ZNF32 promotes GPER expression by binding to the GPER promoter (−1623 bp GCATTT). This finding shows that GPER is a new ZNF32 target gene and induces breast cancer stem cell-like properties by acting upon ERK signalling.

Breast cancer stem cells are also predicted to be responsible for tumour drug resistance. Using Taxol, a breast cancer clinical chemotherapy drug, we showed that GPER, along with ZNF32, is involved in drug sensitivity, as demonstrated by the results of knocking down or over-expressing ZNF32 or GPER. These results suggest that appropriate combination therapies could offer more effective interventions according to the expression pattern of ZNF32 and GPER in breast cancer.

Collectively, our data reveal that ZNF32, via GPER/ERK signalling, is involved in breast cancer stemness and pro-survival effects in cancer cells (Fig. 8). We provide novel insights into the regulation and function of GPER induced by ZNF32 in breast cancer cells. This study also enriches knowledge of the regulatory networks in breast cancer stem cells. We believe that these phenotypes are worthy of additional investigation to further define the functions of ZNF32 and to develop ZNF32-associated targeted therapies. Hopefully, this study will provide another potential way to increase the efficiency of cancer treatment and the overall survival of breast cancer patients.

## Electronic supplementary material

Supplementary Figure 1

Supplementary Figure 2

Supplementary Figure 3

Supplementary Figure 4

Supplementary Table1, 2, 3, 4

Supplementary Figure Legend
