# Supplementary figures and images for: Zinc finger protein 32 promotes breast cancer stem cell-like properties through directly promoting GPER transcription
Source: Cell Death Dis. 2018 Nov 26;9(12):1162. doi: 10.1038/s41419-018-1144-2 (PMC6255875; doi:10.1038/s41419-018-1144-2)

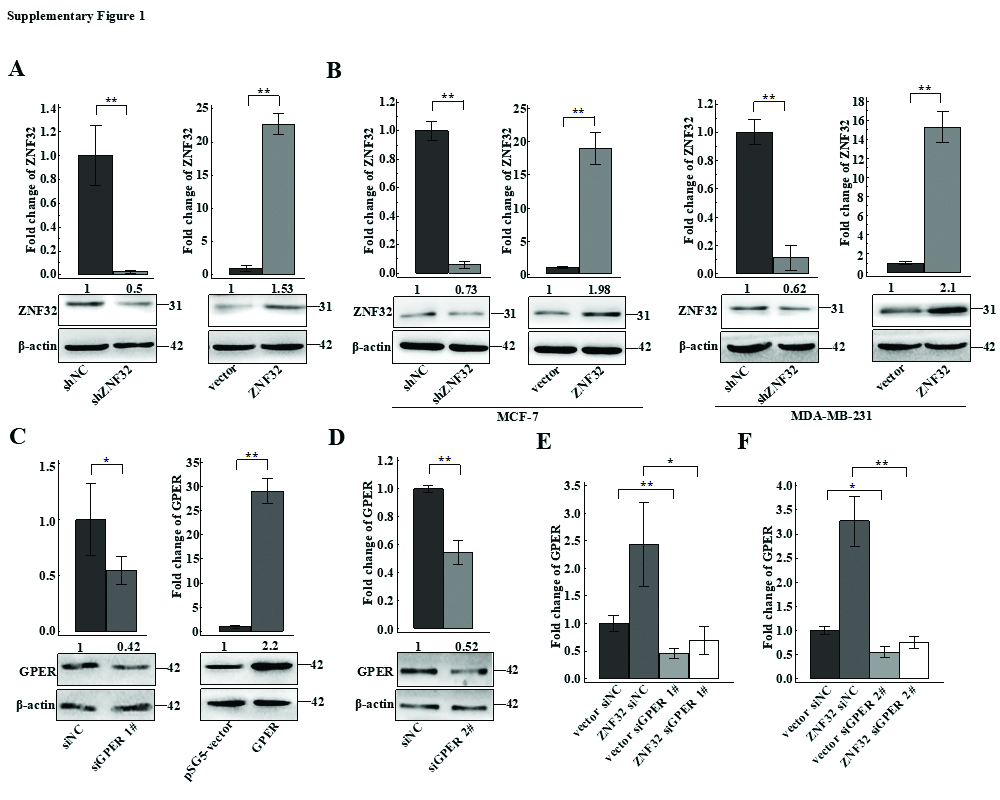

Supplement: Supplementary file 1 — Supplementary Figure 1 [file 41419_2018_1144_MOESM1_ESM.jpg]

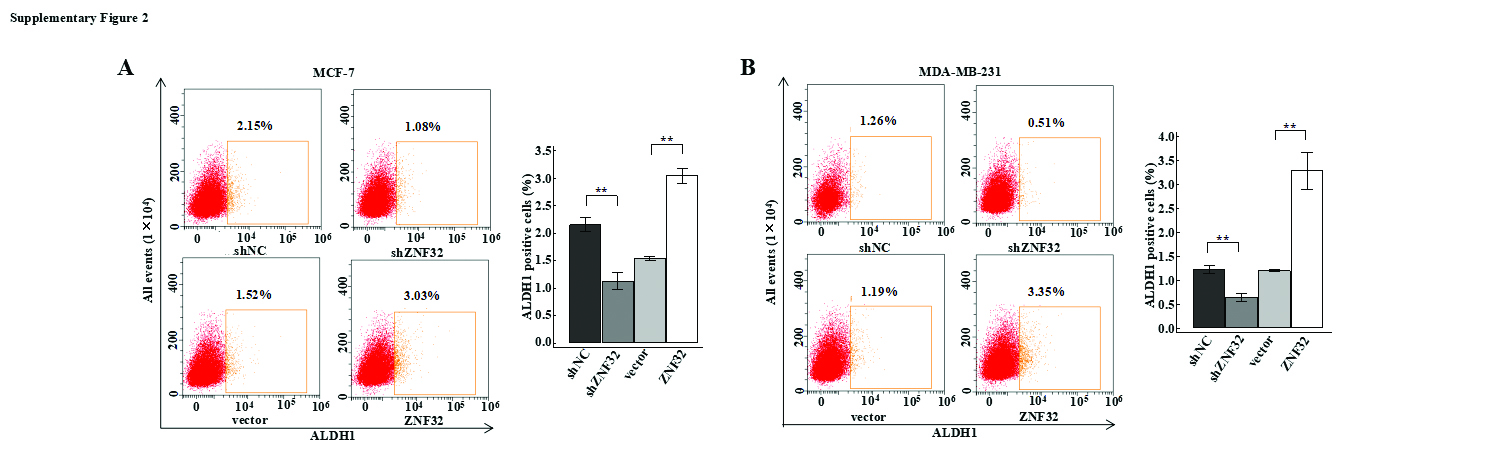

Supplement: Supplementary file 2 — Supplementary Figure 2 [file 41419_2018_1144_MOESM2_ESM.jpg]

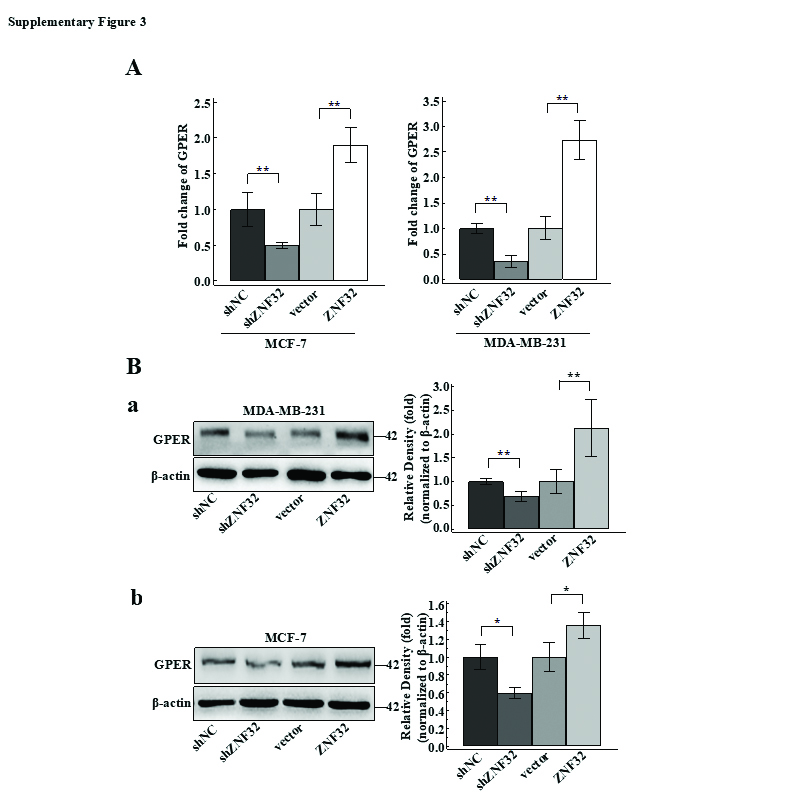

Supplement: Supplementary file 3 — Supplementary Figure 3 [file 41419_2018_1144_MOESM3_ESM.jpg]

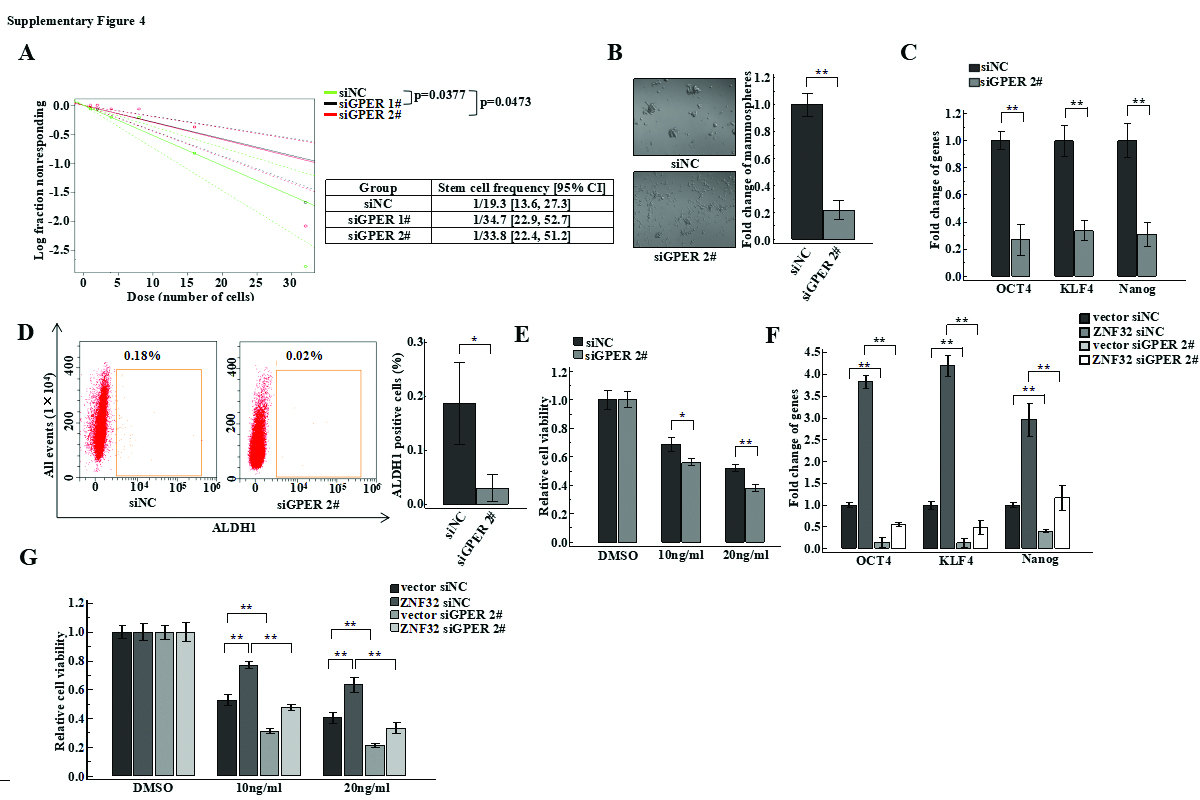

Supplement: Supplementary file 4 — Supplementary Figure 4 [file 41419_2018_1144_MOESM4_ESM.jpg]
