# Supplementary material for: Zinc finger protein 32 promotes breast cancer stem cell-like properties through directly promoting GPER transcription
Source: Cell Death Dis. 2018 Nov 26;9(12):1162. doi: 10.1038/s41419-018-1144-2 (PMC6255875; doi:10.1038/s41419-018-1144-2)
Supplement: Supplementary file 5 — Supplementary Table1, 2, 3, 4 [file 41419_2018_1144_MOESM5_ESM.doc]

Supplementary Table 1. Primers used in this study.

| Gene | Primer sequence (5’--3’) |
| --- | --- |
| ZNF32 | up:GAAGATATGCCCAGAATGTAGCG dw: GGATTTTTGTTCCAGCTTCTCTCT |
| GPER | up:ATGACCATCCCCGACCTGTA dw:TGGTGCTTGGTGCGGAAC |
| OCT4 | up:CAAGCTCCTGAAGCAGAAGAGGAT dw:CTCACTCGGTTCTCGATACTGGTT |
| KLF4 | up:TTCCAAACTGGCGATTCACAA dw:ATTAACTGGCAGAGTGGCAGGTAA |
| Nanog | up:CCGGTCAAGAAACAGAAGACCAGA dw:CCATTGCTATTCTTCGGCCAGTTG |
| GPER ChIP (a) | up:CCATCTCGTAACCCTCTGGA dw: ACCTGGGCATTGTCCA |
| GPER ChIP (b) | up:ACTTTGTGCGTGTTTTTCTCTAT dw:GATCTGATGTCGTTATGAATTTTTA |
| GPER ChIP(c) | up:CATGTCTCAGCTAATTCAAGGT dw:ATGCTGTGTTAATTTATTAATGGA |
| GPER ChIP(GTATTT) | up:TGGGGATGGGACAGGGAGC dw: ATGCACACATAGCCAAATACTGGA |
| GPER ChIP(GCATTT) | up:ATTTGGCTATGTGTGCATTTAGC dw: TCCTTGTTGGATCTGATGATGTCGT |
| β-actin | up: AAGGTGACAGCAGTCGGTTGG dw: GGCAAGGGACTTCCTGTAACAATG |
| pSG5-GPER | up:GGATCCATGGATGTGACTTCCCAAGC  dw:AAGCTTACACGGCACTGCTGAACCT |
| GPER promoter (GCATTT) | up:CGGGGTACCATTTGGCTATGTGTGCATTTAGC  dw:CCGCTCGAGCCTTGTTGGATCTGATGTCGT |
| GPER promoter (GCATTT)-Mut | up:TGGCTATGTGTCCATCTAGCTCAATAAGCA  dw:GCTTATTGAGCTAGATGGACACATAGCCAAAT |

Primers used for pSG5-GPER plasmid construction, qPCR gene amplification, ChIP assay.

Supplementary Table 2. shRNA sequence used in this study.

| Gene | sequence (5’--3’) |
| --- | --- |
| shZNF32 (LV3NC) | GAATGTAGCGTTCTTCAATGT |
| shZNF32 NC (LV3NC) | TTCTCCGAACGTGTCACGT |
| shGPER (LV10NC) | CGAGTTAAAGAGGAGAAGGAA |
| shGPER NC (LV10NC) | TTCTCCGAACGTGTCACGT |

shRNA sequence for lentivious package.

Supplementary Table 3. Double-stranded oligo nucleotide sequence.

| name | Oligos | 5' Mod. | 3' Mod. |
| --- | --- | --- | --- |
| WT ZNF32 site on GPER | Sense: TTC TCT ATG CAT TAT CCA GTA TTT GGC TAT GTG T | biotin | none |
| WT ZNF32 site on GPER | Anti-Sense:ACA CAT AGC CAA ATA CTG GAT AAT GCA TAG AGA A | none | none |
| mt ZNF32 site on GPER | Sense: GGC TAT GTG TCC ATC TAG CTC AAT AAG CAA T | biotin | none |
| mt ZNF32 site on GPER | Anti-Sense: ATT GCT TAT TGA GCT AGA TGG ACA CAT AGC C | none | none |

Double-stranded oligo nucleotide used for Cell extract pulldown assays.

Supplementary Table 4. patient information used in this study.

| case | ER % | PR % | HER2 | Ki-67 % | metastasis | case | ER % | PR % | HER2 | Ki-67 % | metastasis |
| --- | --- | --- | --- | --- | --- | --- | --- | --- | --- | --- | --- |
| 1 | 70 | 40 | 3+ | 30 | yes | 25 | - | - | 1+ | 40 | no |
| 2 | 70 | 70 | 2+ | 10 | yes | 26 | - | - | 1+ | 70 | no |
| 3 | 5 | <1% | 2+ | 10-20 | yes | 27 | <1 | - | 3+ | 30 | no |
| 4 | - | - | 1+ | 40% | no | 28 | - | - | - | 50 | yes |
| 5 | 60 | 70 | 1+ | No | yes | 29 | 70 | 90 | 1+ | 40 | no |
| 6 | - | - | 1+ | 15-20 | yes | 30 | Mode | - | 2+ | 50-60 | yes |
| 7 | - | - | 2+ | 40-50 | yes | 31 | 90 | 90 | - | 5-10 | no |
| 8 | - | - | 3+ | 30 | no | 32 | 90 | 90 | 0+ | 5-10 | no |
| 9 | >90 | 80 | Mode | 2-6 | no | 33 | 90 | 5-10 | 2+ | 10 | no |
| 10 | >90 | 80 | 1+ | 5-15 | no | 34 | - | - | 1+ | 30 | no |
| 11 | - | - | 3+ | 30 | yes | 35 | - | - | 3+ | 50 | no |
| 12 | - | - | 2+ | 15 | yes | 36 | - | Mode | No | 20-30 | no |
| 13 | 90 | 90 | 0+ | 5-10 | no | 37 | <1 | 60 | No | 30-40 | no |
| 14 | - | - | 2+ | 30 | no | 38 | - | - | No | 40-50 | no |
| 15 | 60 | 5 | 3+ | 5-10 | yes | 39 | >90 | 70 | 2+ | 10-20 | yes |
| 16 | 50 | 5 | 1+ | 40 | no | 40 | >95 | 60 | 1+ | 30-40 | no |
| 17 | - | - | 3+ | 30 | no | 41 | 50-60 | 70 | 2+ | 15-20 | no |
| 18 | 1 | <1 | 3+ | 30-40 | no | 42 | - | - | 1+ | 15-20 | yes |
| 19 | 20 | 30 | 2+ | 40 | yes | 43 | - | - | 1+ | 15-20 | yes |
| 20 | 60 | 80 | 1+ | 10 | no | 44 | 60 | 60 | 1+ | 10 | no |
| 21 | 10 | >70 | 2+ | 30 | no | 45 | 60 | 60 | 1+ | 10 | no |
| 22 | - | - | 3+ | 50 | no | 46 | - | - | 1+ | 50 | yes |
| 23 | - | - | 1+ | 30 | yes | 47 | - | <1 | 3+ | 30-40 | no |
| 24 | - | - | 2+ | 15 | yes | 48 | - | <1 | 3+ | 30-40 | no |

patient information for experiment.
