# Supplementary material for: Zinc finger protein 32 promotes breast cancer stem cell-like properties through directly promoting GPER transcription
Source: Cell Death Dis. 2018 Nov 26;9(12):1162. doi: 10.1038/s41419-018-1144-2 (PMC6255875; doi:10.1038/s41419-018-1144-2)
Supplement: Supplementary file 6 — Supplementary Figure Legend [file 41419_2018_1144_MOESM6_ESM.doc]

**Figure S1.** (A) (B) Western blotting analysis (lower panels) and qPCR (upper panels) were used to confirm knockdown and over-expression efficiency of ZNF32 in ZR-75-30, MCF-7 or MDA-MB-231 cells. (C) Western blotting analysis (lower panels) and qPCR (upper panels) were used to confirm knockdown and over-expression efficiency of GPER in ZR-75-30 cells. (D) Western blotting analysis (lower panels) and qPCR (upper panels) were used to confirm knockdown efficiency of siGPER 2# in ZR-75-30 cells. (E) qPCR analysis was used to confirm interference efficiency of siGPER 1# in ZNF32 over-expression ZR-75-30 cells. (F) qPCR analysis was used to confirm interference efficiency of GPER by siGPER 2# in ZNF32 over-expression ZR-75-30 cells.

**Figure S2.** (A) (B) ALDH1-positive cells were detected by flow cytometry using an ALDH1^+^ gate based on shNC or vector cells. The bar graph in the right panel shows the number of ALDH1-positive cells in the shNC, shZNF32, vector, and ZNF32 MCF-7 and MDA-MB-231 cells.

**Figure S3.** (A) qPCR analysis of *GPER, ERα* and *ERβ* levels in ZNF32 knockdown or over-expressing MCF-7 and MDA-MB-231 cells. (B) Western blotting was used to confi­rm the effect of ZNF32 knockdown or over-expression on GPER expression in MDA-MB-231 (a) and MCF-7 (b) cells, and the right panels show the quantification of 3 independent experiments.

**Figure S4.** (A) Stem cell frequency was calculated using the online Extreme Limiting Dilutions Assay (ELDA) analysis program. A significant difference in stem cell frequency was detected between siNC (1/19.3), siGPER 1# (1/34.7) and siGPER 2# (1/33.8) ZR-75-30 cells. (B) Representative images of mammosphere formation, and bar graph showing the fold change in the number of mammospheres per field in the siNC, siGPER 2#. siGPER 2# groups compared to the siNC groups. (C) *OCT4*, *KLF4* and *Nanog* expression levels were detected by qPCR in GPER knockdown ZR-75-30 cells. (D) ALDH1-positive cells were detected by flow cytometry using an ALDH1^+^ gate based on siNC or siGPER 2# cells. The bar graph in the right panel shows the number of ALDH1-positive cells in the siNC and siGPER 2# ZR-75-30 groups. (E) ZR-75-30 cells with GPER knockdown were plated in 96-well plates and incubated for 24 h, and then, 10 ng/ml or 20 ng/ml Taxol or DMSO as a solvent control was added. MTT analysis was used to assess cell viability. (F) *OCT4*, *KLF4* and *Nanog* gene expression were measured by qPCR in ZNF32 over-expressing ZR-75-30 cells with or without GPER knockdown. (G) ZR-75-30 cells with ZNF32 over-expression were plated in 96-well plates, and GPER expression was knocked down using siRNA. After 24 h of incubation, 10 ng/ml or 20 ng/ml Taxol or DMSO (control) was added, and MTT analysis was used to detect cell viability.
